# Effect of post-mastectomy radiation therapy on survival in breast cancer with lymph nodes micrometastases: a meta-analysis and systematic review

**DOI:** 10.3389/fonc.2025.1489390

**Published:** 2025-05-08

**Authors:** Jianqing Zheng, Bifen Huang, Ying Chen, Zhangzhu Chen

**Affiliations:** ^1^ Department of Radiation Oncology, The Second Affiliated Hospital of Fujian Medical University, Quanzhou, Fujian, China; ^2^ Department of Obstetrics and Gynecology, Quanzhou Medical College People’s Hospital Affiliated, Quanzhou, Fujian, China; ^3^ Department of Emergency, The Second Affiliated Hospital of Fujian Medical University, Quanzhou, Fujian, China

**Keywords:** lymph nodal micrometastasis, radiotherapy, breast cancer, sentinel lymph node biopsy, axillary lymph node dissection, meta-analysis

## Abstract

**Background:**

Axillary management of patients with early-stage breast cancer (ESBC) has evolved, especially with the implementation of precision radiotherapy techniques that have resulted in a significant reduction in treatment-related toxicities, but it is unclear whether post-mastectomy radiotherapy (PMRT) improves survival outcomes in breast cancer with lymph nodes micrometastases (BCLNMM, that is T0, T1 ~2NmiM0). Our study is to systematically evaluate the effect of PMRT on survival in breast cancer with lymph nodes micrometastases.

**Methods:**

A literature search was performed for randomized controlled trials (RCTs) or retrospective studies related to PMRT versus non-post-mastectomy radiotherapy (non-PMRT) in the adjuvant treatment of ESBC in PubMed, Cochrane Library, Embase, CNKI and other databases. R package *meta* software was used to perform meta-analyses with hazard ratio (HR). Newcastle Ottawa scale was selected for quality assessment. The review was prospectively registered on PROSPERO (CRD42024562444).

**Results:**

10 relevant studies were screened, all of which were retrospective studies. The difference in overall survival (OS) was not statistically significant (HR = 0.92, 95%CI: 0.81 ~ 1.04; Z = 1.35, *P* = 0.177). The difference in breast cancer-specific survival (BCSS) between the PMRT group and the non-PMRT group was not statistically significant HR = 1.18, 95%CI: 0.94 ~ 1.48; Z = 1.41, *P* =0.160). The difference in disease-free survival (DFS) was statistically significant (HR = 0.47, 95%CI: 0.23 ~ 1.00; Z = 1.96, *P* =0.049). The difference in local recurrence free survival (LRRFS) was also not statistically significant (HR = 0.50, 95%CI: 0.11 ~ 2.26, *P* = 0.190). The difference in distant-metastasis free survival (DMFS) was not statistically significant (HR = 0.54, 95%CI: 0.22 ~ 1.35, *P* = 0.356).

**Conclusions:**

Despite the tendency of PMRT in BCLNMM to improve DFS, OS, BCSS, LRRFS, and DMFS showed no benefit, therefore, PMRT should be used with caution in BCLNMM.

**Systematic review registration:**

https://www.crd.york.ac.uk/prospero/, identifier CRD42024562444.

## 1 Introduction

Breast cancer (BC) has currently replaced lung cancer as the most commonly diagnosed cancer in humans worldwide (1, 2). According to GLOBOCAN 2020 data, an estimated number of 2.3 million new BC cases were diagnosed worldwide, which leads the 5th cause of cancer-related deaths among all cancers (3). In China, the annual number of new cases of BC reaches 420,000 and the number of deaths reaches 120,000, accounting for 18.4% and 17.1% of the global cases, respectively (4). Although the incidence of breast cancer is lower than that of lung cancer in China, the absolute number of cases is still the highest in the world (5). In recent years, with the development of early detection, early diagnosis, precise typing and adjuvant treatment (chemotherapy, endocrine therapy, targeted therapy, radiation therapy) of BC, the prognosis of early-stage BC patients has improved significantly compared with the former (6).

The presence or absence of axillary lymph node metastasis (LNM) is an important index to evaluate the risk of breast cancer recurrence. According to the eighth edition of the Cancer Staging Manual of the American Joint Committee on Cancer (AJCC), N stages of BC were divided into large metastases (larger than 2mm), micro-metastases (N1mi, 0.2-2mm metastasis) and isolated tumor cells (ITC, single tumor cells or small cell clusters not larger than 0.2mm, pN0(i+)) according to lymph node involvement (7). Some previous studies have supported the independent prognostic significance of LNM, and the prognosis was worse than that of lymph nodes without metastasis (8, 9).

Lymph node micrometastases (LNMM) was defined as lymph node metastases less than 2mm in size, which is the first pathological manifestation of distant metastasis of breast cancer (10). With the development of comprehensive treatment in BC, surgical treatment has gradually become more precise and less invasive (11). Sentinel lymph node biopsy (SLNB) has been widely recognized as an excellent surgical and staging procedure for early-stage BC, and is now routinely used to detect the status of axillary lymph node metastases in BC and as a basis for selecting subsequent adjuvant therapy (12, 13). The development of SLNB has greatly improved the detection of micrometastases (14). When LNMM was detected in sentinel lymph nodes, it indicates that cancer cells had spread to the lymphatic system (15). However, because the degree of metastasis is limited, the following axillary treatment of micrometastasis has been the subject of much debate (16). The diagnostic and treatment options and risk of recurrence vary among patients with different SLN metastatic status. Micrometastasis to sentinel lymph nodes is associated with poor disease-free survival, and it is generally accepted that patients with micrometastasis to sentinel lymph nodes should be treated with postoperative adjuvant chemotherapy after total mastectomy (10, 17). Although systemic chemotherapy is effective in improving overall survival, local or regional recurrence remains the most common model of failure for breast cancer, meaning that local treatment may be necessary (18). Therefore, more and more attention has been paid to the local treatment of LNMM patients to further improve the therapeutic effect (11). The main local adjuvant treatment for breast cancer patients with positive lymph node micrometastases includes radiation therapy or further axillary lymph node dissection, while the value of these two treatments is still widely debated (12).

With the continuous development of precision radiotherapy, the damage caused by radiotherapy is becoming less and less, which makes it possible to replace regional lymph node dissection with radiotherapy (19). Breast cancer surgery currently focuses on reducing the intensity of treatment and the scope of surgery without compromising patient survival (19). Postmastectomy radiotherapy (PMRT) is currently sufficient to replace axillary lymph node dissection (ALND) in patients with less than 3 sentinel lymph nodes (SLNs) involvement in primary surgery, which not only significantly reduces the incidence of lymphedema, but also does not reduce the long-term prognosis of breast cancer (20). However, in most cases, the incidence of residual axillary disease is low despite the risk of micrometastasis in axillary lymph nodes, which results in patients with LNMM not benefiting from postoperative axillary radiotherapy (ART). Data on the use of PMRT with a view to improving long-term survival in this population is extremely scarce, and there are currently no randomized controlled studies supporting evidence for PMRT. In this paper, we used Meta-analysis to study the existing literature to investigate whether PMRT is necessary for early-stage breast cancer patients with micrometastases in the sentinel lymph nodes (T0, T1-2NmiM0) after mastectomy, with the aim of systematically evaluating the safety and efficacy of PMRT in patients with micrometastases in the sentinel lymph nodes, and to provide a basis for the formulation of clinical treatment plans.

## 2 Materials and methods

### 2.1 Study protocol

The current study was conducted according to the Preferred Reporting Items for Systematic Reviews and Meta-analyses (PRISMA) (21), and the quality control and quality assurance (QC & QA) of the manuscript was instructed by the corresponding authors (Jianqing Zheng and Zhangzhu Chen). The review was prospectively registered on PROSPERO (CRD42024562444).

### 2.2 Literature inclusion criteria

#### 2.2.1 Study design

If possible, randomized controlled trials (RCTs) will be preferred. In the absence of high-quality RCTs, well-designed retrospective studies may also be considered for inclusion in the final systematic review.

#### 2.2.2 Study participants

(1) Study participants were BC patients diagnosed and confirmed by pathology; (2) were diagnosed as BC patients with sentinel lymph node micrometastasis (T0, T1 ~ 2NmiM0); (3) were the patients who have not received any other pre-surgery treatments in the past, including chemotherapy, targeted therapy, etc.

#### 2.2.3 Interventions

(1) Interventions were conventional chemotherapy in the control group. However, the chemotherapy regimen and chemotherapy cycle were not limited. (2) Interventions were PMRT in the experimental group, and other clinical treatments were the same as those in the control group.

#### 2.2.4 Outcomes

(1) The primary outcomes are overall survival (OS) and breast cancer-specific survival (BCSS). (2) The secondary outcomes were disease-free survival (DFS), local recurrence free survival (LRRFS) and distant-metastasis free survival (DMFS).

### 2.3 Literature exclusion criteria

The exclusion criteria included: (1) Studies involving non-clinical trials or single arm trials; (2) Research with incomplete data or the relevant data could not be extracted. (3) Repeated publications. If serial clinical studies had the most recent literature, the most recent data were included in the analysis. (4) Studies involving patients that received any other pre-surgery treatments in the past, such as chemotherapy, molecular targeted therapy, etc.

### 2.4 Search strategies

#### 2.4.1 Database

A comprehensive literature search on the PubMed Database, Embase Database, Web of science (WOS) database, Cochrane Library, China Biomedical Literature Database (CBM), China National Knowledge Database (CNKI), and Wanfang Database were performed, covering all publications in these databases up to February 1^st^, 2024. As examples, search strategies for PubMed and Embase were listed in **Supplementary Data Sheet 1**.

#### 2.4.2 Search terms

(1) Search terms related to disease were breast cancer, breast neoplasm, breast carcinoma, breast tumor, etc. (2) Search terms related to radiotherapy were radiotherapy, radiation therapy, intensity modulated radiation therapy, three-dimensional conformal radiation therapy, etc. (3) Other search terms related to disease status included lymph node micrometastasis, localized lymph node metastasis, etc.

#### 2.4.3 Retrieval strategies

The subject terms with free words were applied to conduct a preliminary retrieval of the literatures in the above database. After a detailed screening of the literature, reviews, case reports, meta-analysis and other types of literatures were filtered out by Endnote software. Independent searches were conducted by 2 investigators (Jianqing Zheng and Bifen Huang) in accordance with the above search principles. 2 investigators (Ying Chen and Jianqing Zheng) further evaluated and confirmed the included studies. When there was a disagreement, the third investigator (corresponding author) will be consulted. Further manual and electronic database searches were carried out through the reference lists attached to the eligible articles. At the same time, search engines such as Google Scholar or Baidu Scholar were used to find relevant literatures on the Internet, and to trace the references that had been included in the literature, in order to expand the scope of retrieval.

### 2.5 Literature extraction and quality assessment

#### 2.5.1 Literature extraction

Two independent researchers (Jianqing Zheng and Bifen Huang) reviewed and evaluated the title and abstract of each trial according to the determined search strategies, and the potentially eligible articles that meet the selection criteria would be recruited. After discussion in accordance with the inclusion criteria was performed and a consensus was reached, a decision was made to finally include or exclude the eligible articles. If a consensus couldn’t be met, the corresponding author (Zhangzhu Chen) of this article was responsible for the final ruling.

#### 2.5.2 Quality assessment

Two reviewers (Jianqing Zheng and Bifen Huang) were responsible for independent literature quality evaluation, the database was established, and the inconsistencies were discussed and resolved through negotiation. RCTs were assessed according to the bias risk assessment method recommended by the Cochrane Assistance Network (22), while non- RCTs were assessed by the Newcastle-Ottawa Scale (NOS) (23). The evaluation methodological criteria and items for RCTs were as follows: (1) Generation of random allocation sequence; (2) the method of allocation concealment; (3) the method of blinding the patients; (4) the method of blinding the doctors or the therapists; (5) the method of blinding the data collectors and analysis personnel; (6) incomplete data reported; (7) selective reporting bias; (8) other potential bias affecting authenticity. We evaluate the risk of bias for each RCT according to the following criteria: “Yes” indicates a low risk of bias; “No” indicates a high risk of bias; “Unclear” indicates that the literature does not provide sufficient information for bias assessment. The NOS is divided into 8 items with a full score of 9, including selection of population (4 items with a total score of 4), comparability between groups (1 item with a total score of 2), and measurement of exposure factors (3 items with a total score of 3) (23). The total score 0–4 for all NOS items was classified as low-quality studies, and 6–7 as moderate-quality studies, and 8–9 as high-quality studies.

During the literature search process, two reviewers (Jianqing Zheng and Bifen Huang) were responsible for searching and tracking the references of important literature if necessary.

### 2.6 Data extraction

After reading the full text, two researchers extracted and cross-checked the data, including: (1) Basic information: such as title of the trial, author’s name, year of publication, source of literature, and other features; (2) Methodological information of the trial: Study design type, the sample size of the study included, the basic information of the study population, including the entry time and number of participants, disease stages, etc.; the evaluation method of important outcome indicators; median follow-up duration, death and withdrawal, etc. (3) Detailed information on intervention measures: detailed information on radiation therapy, medication in the control group, etc. (4) Outcome indicators: HR for all survival indicators with corresponding 95% CIs and standard errors. Disagreements were resolved by consensus.

### 2.7 Statistical analysis

For survival measures, HR and its corresponding 95% CIs were used as the effect size. If HR and its 95% CIs cannot be obtained directly from the trials, they are extracted according to the method introduced by Parmar et al. (24). Quantitative and comprehensive analysis was performed using R-package *meta*. Chi-square test (χ^2^ test) was used to determine whether there was heterogeneity within the study, and index I^2^(range 0 ~ 100%) was used to measure the degree of heterogeneity. The I^2^ index ≥50% or the *P* value <0.1 of the χ^2^ test indicated significant heterogeneity among studies. If there is no statistical heterogeneity or low statistical heterogeneity, the fixed effects model would be used for combine survival indicators. If not, a random effects model would be used for meta-analysis. Publication bias was analyzed by funnel plot. The source of heterogeneity was evaluated by sensitivity analysis. *P* < 0.05 was considered statistically significant.

## 3 Results

### 3.1 Literature search results

**Figure 1:**
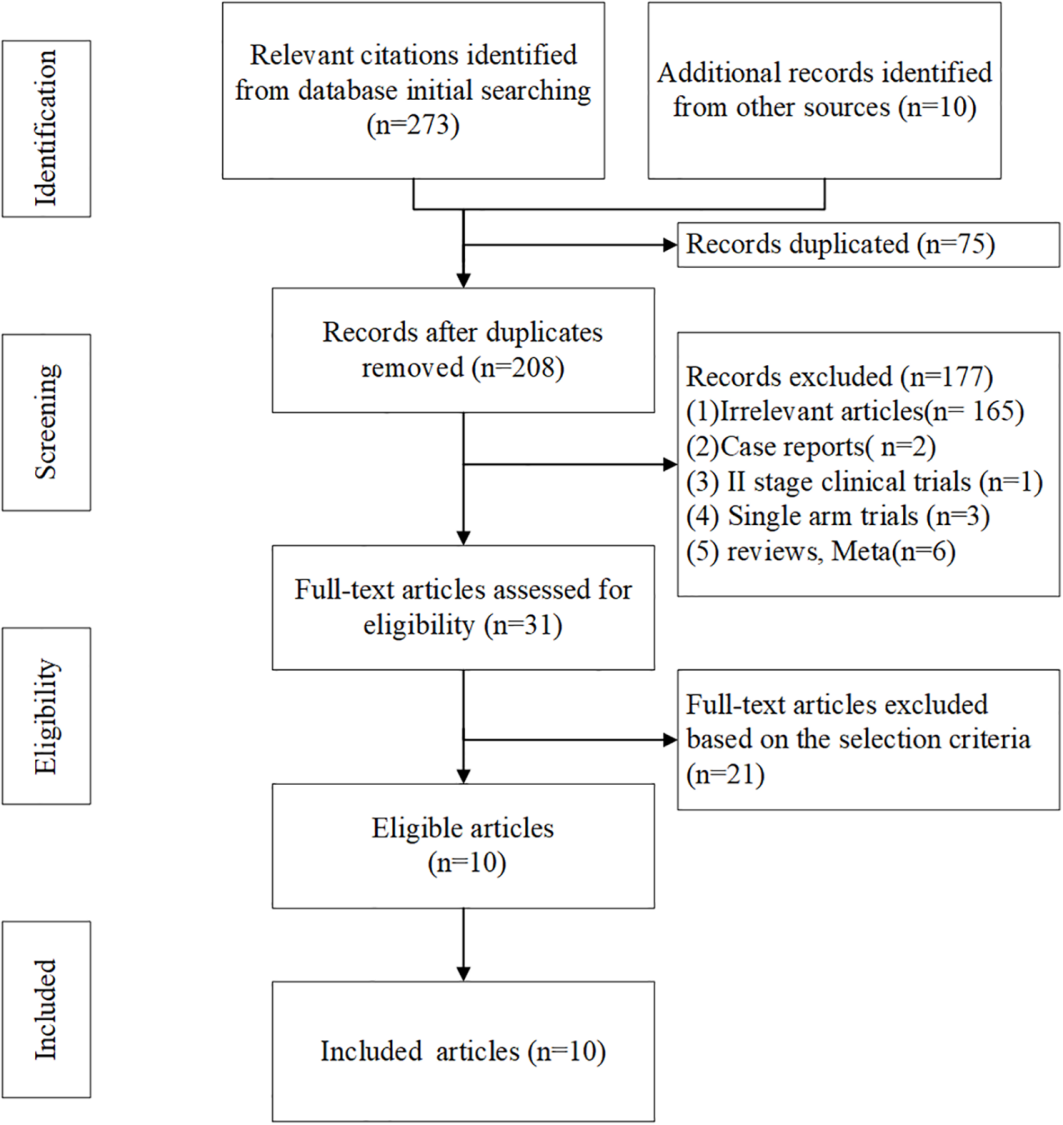
Trials selection flow chart of the meta-analysis.

283 literatures were initially retrieved from domestic and foreign databases, among which 75 were identified as duplicates and then removed. 177 literatures were removed after reading the title and abstract of the literatures because of irrelevant features, case reports, II stage clinical trials, etc. 21 literatures were excluded from intervention measures or subjects did not conform to our study purpose. So, 10 literatures were finally included (16, 25–33). The literature screening process was shown in **Figure 1**.

All 10 studies were retrospective and no prospective randomized controlled studies were available. The included studies were published in English, with a median follow-up of 30 to 98 months. A total of 76905 patients were included. At the same time, publication year, the grouping detail, total number of cases, outcome indicators, follow-up time of the 10 literatures were summarized in **Table 1**.

**Table 1:** Basic features of included studies.

Author	Year	Sample size	PMRT	Non-PMRT	Median Follow-Up	Outcomes
Chang (25)	2015	120	20	100	98	LRRFS, DMFS, DFS, OS
Eastwick (26)	2018	3242	NA	NA	NA	OS
Picado (27)	2018	17051	NA	NA	53	OS
Wu (28)	2018	14326	2651	11368	53.1	OS
Zhang (29)	2019	1571	254	1317	30	OS, BCSS
Patel (30)	2020	5878	1202	4676	NA	OS
Shi (31)	2020	4729	1062	3667	49	BCSS
Lim (32)	2021	92	31	61	60	DFS, OS
Luo (33)	2022	2864	588	2276	53	BCSS
Luo (16)	2023	27032	14368	12664	NA	OS

NA, Not available; LRRFS, local recurrence free survival; DMFS, distant-metastasis free survival; OS, overall survival; BCSS, breast cancer-specific survival; DFS, disease-free survival.

### 3.2 Quality assessment of included studies

Methodological quality evaluation was carried out on the included studies, and the scores of all studies were high, as shown in **Table 2**.

**Table 2:** The Newcastle-Ottawa Scale (NOS) checklist of included studies.

Author	Year	Cohort selection				Comparability	Outcome ascertainment			Scores
		Representativeness of the Exposed Cohort	Selection of the NonExposed Cohort	Ascertainment of Exposure	Demonstration that Outcome of Interest Was Not Present at Start of Study	Comparability of Cases and Controls on the Basis of the Design or Analysis	Assessment of Outcome	Was FollowUp Long Enough for Outcomes to Occur	Adequacy of Follow Up of Cohorts	
Chang (25)	2015	1	1	1	1	1	1	1	1	8
Eastwick (26)	2018	1	1	1	1	1	1	0	0	6
Picado (27)	2018	1	1	1	1	1	1	1	1	8
Wu (28)	2018	1	1	1	1	2	1	1	1	9
Zhang (29)	2019	1	1	1	1	1	1	1	1	8
Patel (30)	2020	1	1	1	1	1	1	0	1	7
Shi (31)	2020	1	1	1	1	1	1	1	1	8
Lim (32)	2021	1	1	1	1	1	1	1	1	8
Luo (33)	2022	1	1	1	1	2	1	1	1	9
Luo (16)	2023	1	1	1	1	2	1	1	1	9

### 3.3 Meta-analysis of overall survival

**Figure 2:**
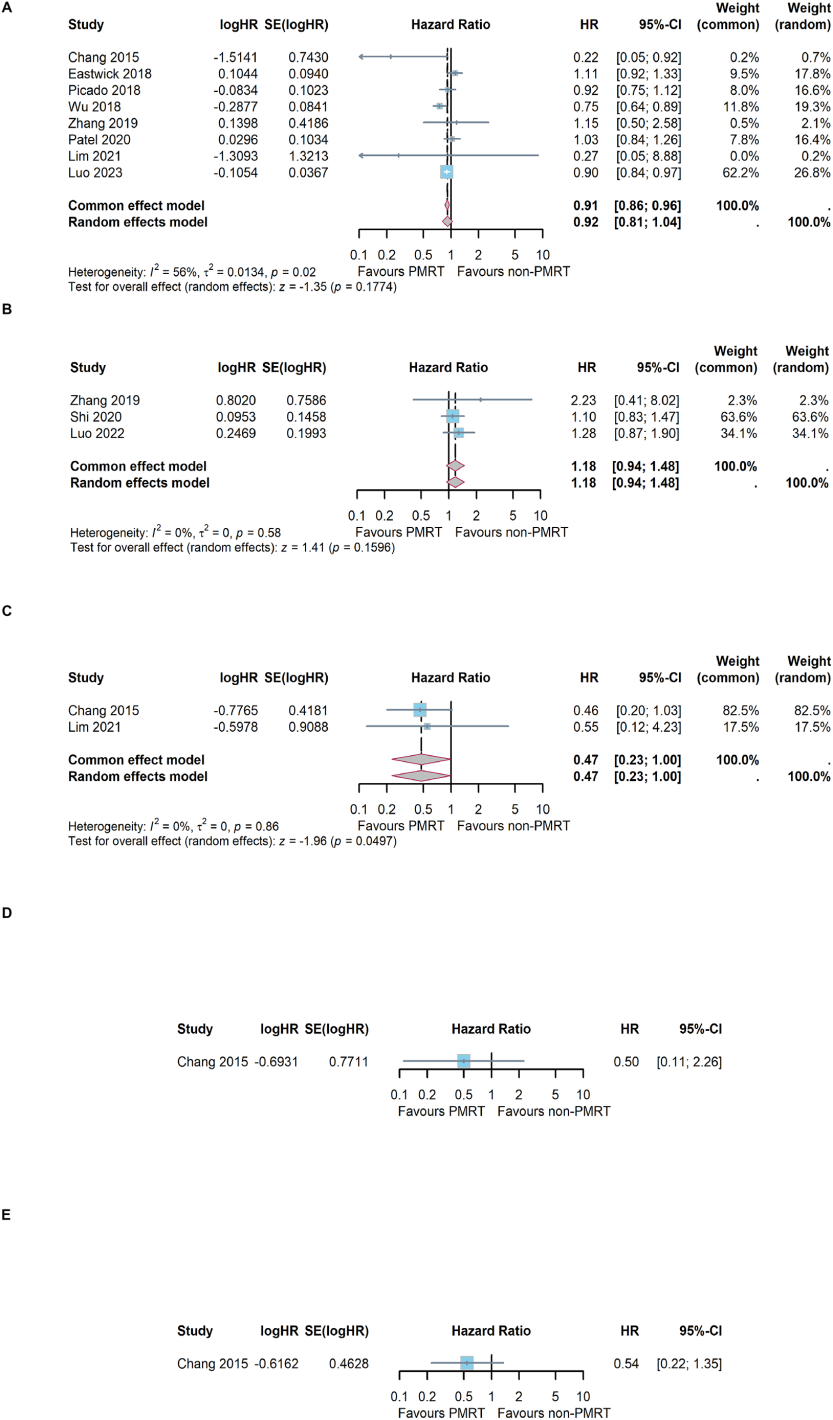
Meta-analysis results of different survival outcome. **(A)** overall survival, **(B)** breast cancer-specific survival, **(C)** disease-free survival, **(D)** local recurrence free survival and **(E)** distant-metastasis free survival.

Among the included literatures, 8 trials had reported OS results in terms of effect index HR (16, 25–30, 32). The result of heterogeneity test was I^2^ = 56%, and *P* = 0.02 with Q tests, indicating that there is moderate heterogeneity among the literatures included in this study. Therefore, a random effect model was selected for meta-analysis. The results showed that the combined effect size HR = 0.92, 95%CI: 0.81 ~ 1.04; Z = 1.35, *P* = 0.177, as shown in **Figure 2A**. The results indicated that PMRT did not improve OS in patients with sentinel lymph node micrometastasis who underwent mastectomy.

### 3.4 Meta-analysis of breast cancer-specific survival

Among the included literatures, 3 trials had reported BCSS results (29, 31, 33). The result of heterogeneity test was I^2^ = 0%, and *P* = 0.58 with Q tests, indicating that there is no heterogeneity among the included trials. Therefore, a fixed effect model was selected for meta-analysis. The results showed that the combined effect size HR = 1.18, 95%CI: 0.94 ~ 1.48; Z = 1.41, *P* =0.160, as shown in **Figure 2B**. The results indicated that PMRT did not improve BCSS in patients with sentinel lymph node micrometastasis who underwent mastectomy.

### 3.5 Meta-analysis of disease-free survival

A total of 2 of the 10 included studies had reported the DFS results (25, 32). The heterogeneity test showed that I^2^ = 0% and *P*=0.86, indicating that the included studies were of good homogeneity, and the fixed effect model should be used. Meta-analysis effect size of DFS was HR = 0.47, 95%CI: 0.23 ~ 1.00; Z = 1.96, *P* =0.049, as shown in **Figure 2C**. The results indicated that PMRT could improve DFS in patients with sentinel lymph node micrometastasis who underwent mastectomy.

### 3.6 Meta-analysis of local recurrence free survival and distant-metastasis free survival

Only 1 trial had reported the LRRFS results (25). The effect size of LRRFS was HR = 0.50, 95%CI: 0.11 ~ 2.26, *P* = 0.190, as shown in **Figure 2D**. Only 1 trial had reported the DMFS results (25). The effect size of DMFS was HR = 0.54, 95%CI: 0.22 ~ 1.35, *P* = 0.356, as shown in **Figure 2E**.

### 3.7 Analysis for publication bias

**Figure 3:**
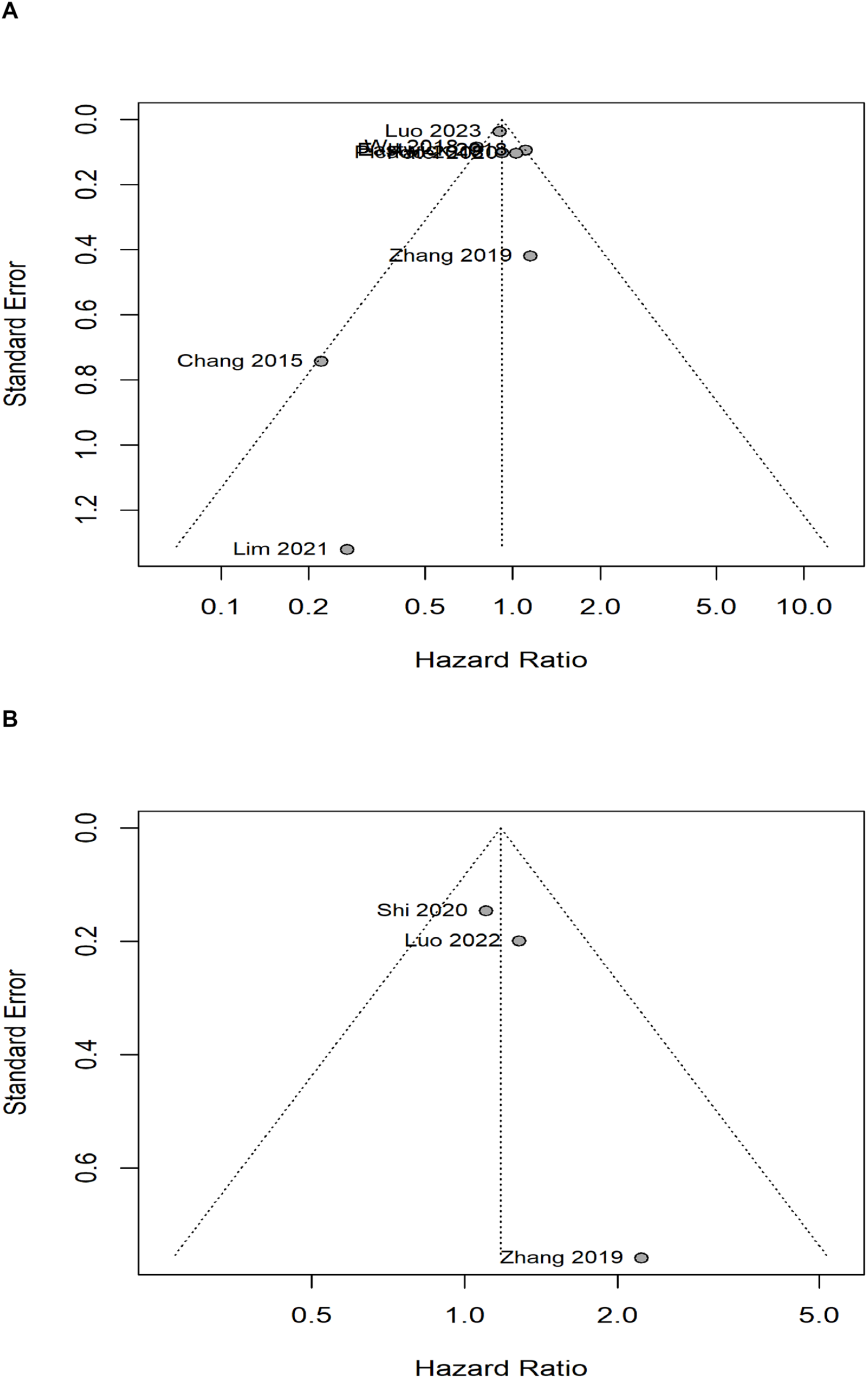
Analysis of publication bias with bias funnel plots. **(A)** overall survival, **(B)** breast cancer-specific survival.

The publication bias funnel plots for OS and BCSS were shown in **Figures 3A, B**. The Egger’s test suggests that the funnel plots for OS (t= 0.44, *P*=0.678) and BCSS (t= 2.73, *P*= 0.224) were basically symmetric, indicating no publication bias. For DFS, LRRFS and DMFS, publication bias analysis was not possible due to the insufficient number of included studies.

### 3.8 Sensitivity analysis of overall survival and breast cancer-specific survival

**Figure 4:**
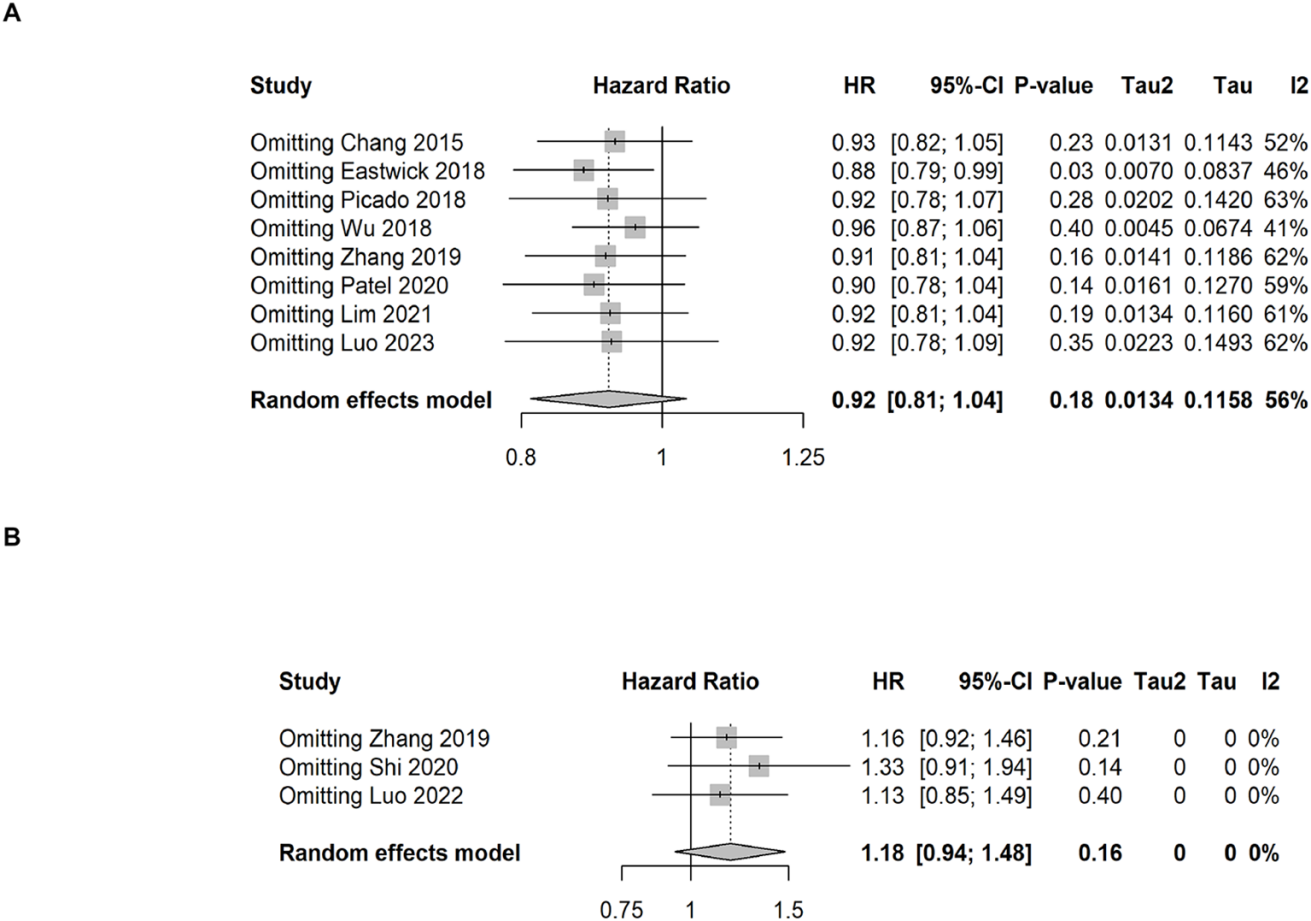
Sensitivity analysis results of overall survival and breast cancer-specific survival. **(A)** overall survival, **(B)** breast cancer-specific survival.

The sensitivity analysis for OS and BCSS were shown in **Figures 4A, B**. Regardless of OS or BCSS, the random exclusion of the included literature did not significantly affect the meta-analysis results, indicating that the results of the random effects model in this study have high stability and reliability.

## 4 Discussion

With the innovative development of pathological diagnostic techniques, sentinel lymph node micrometastases have been detected in more and more breast cancers (34). Although it has been proved that lymph node micrometastasis of breast cancer may be associated with poor prognosis (35), there is a lack of high-quality clinical evidence on whether these patients need to receive local treatment after surgery (36). Compared with axillary lymph node dissection, postoperative axillary radiotherapy can effectively reduce the psychological trauma caused by the second operation and the injury of axillary lymphedema (37). This has led to more and more attempts to improve the prognosis of breast cancer patients with lymph node micrometastases through postoperative radiotherapy (38). However, as far as we know, there is no high-quality evidence of large scale RCTs to support that PMRT can obtain significant clinical benefits, which suggests that it is very necessary to obtain high-quality research evidence through meta-analysis. In our present systematic review, we pooled 10 published retrospective studies in a meta-analysis and found no survival benefit from PMRT in breast cancer patients with lymph node micrometastases. Although limited by the lack of access to RCTs, our meta-analysis process is scientifically sound, prompting us to further consider the value and rationality of postoperative radiotherapy.

Previous clinical practice has shown that axillary lymph node dissection (ALND) is a remedial treatment for early-stage sentinel lymph node micrometastatic breast cancer (39). The rapid development of intraoperative pathologic diagnostic techniques also avoids the risk of a second operation. However, multiple previous randomized controlled trials have shown that further axillary lymph node dissection does not improve overall survival of early-stage sentinel lymph node micrometastatic breast cancer, or even reduce the rate of local recurrence and distant metastasis (39–41). In the IBSCG-23–01 study, breast cancer patients with stage T1 and T2 and clinically inaccessible axillary sentinel lymph nodes with one or more micrometastases were divided into ALND and non-ALND groups, and after 10 years of follow-up, they were showed no difference in axillary recurrence rate and DFS between the two groups (39). A retrospective propensity matching study based on the SEER database of the National Cancer Institute also found that 427,131 patients with TxN1miM0 breast cancer, who were divided into ALND and non-ALND groups, also showed no difference in overall survival between the two groups (12). Therefore, based on the available evidence, breast cancer patients with lymph node micrometastases are not suitable for further axillary lymph node dissection.

As one of the most important non-surgical treatments to get better local tumor control, PMRT is widely used in locally advanced breast cancer or breast cancer with more than 3 lymph node metastases. A large meta-analysis conducted by Early Breast Cancer Trialists’ Collaborative Group (EBCTCG) have shown that in patients with pN+ disease, PMRT reduced the 10-year recurrence risk from 63.7% to 42.5% (*P*<0.001) and the 15-year risk of breast cancer death from 51.3% to 42.8% (P=0.01) (42). Postoperative radiotherapy has higher economic benefits and less toxic effects than secondary surgery, which is more significant in breast cancer patients with lymph node macrometastases (43). For breast cancer patients with 1–3 positive lymph nodes, another large meta-analysis conducted by the EBCTCG, showed a significant improvement in LRR rates among patients receiving PMRT (44). Most current RCTs demonstrated a trend towards OS improvement, while only few studies showed a statistically significant OS or BCSS benefit for PMRT (44). However, there is still no uniform conclusion on whether PMRT is necessary for breast cancer patients with lymph node micrometastases (45). We performed the current review of the literature that focuses on the effect of PMRT in breast cancer patients with lymph node micrometastases, mainly examining several important survival outcomes. As we found, only a trend towards DFS improvement was found in our meta-analysis, with HR = 0.47, 95%CI: 0.23 ~ 1.00; Z = 1.96, *P* =0.049. However, for other survival outcomes, such as OS, BSCC, LRRFS, and DMFS, postoperative radiotherapy did not confer any survival benefit. Although a small number of studies found that postoperative radiotherapy improved OS, the weight of these studies was low in the meta-analysis, and sensitivity analysis showed that these studies did not change the final conclusions (16, 25, 28).

It is worth noting that patients in study of Lim et al. were BC receiving sentinel lymph node biopsy without axillary lymph node dissection after lumpectomy, and about two-thirds of them did not receive PMRT (32). However, they found that DFS and OS were not significantly affected whether PMRT was applied. Lim et al. concluded that PMRT should be avoided in these patients with sentinel lymph node micrometastases who underwent mastectomy with or without axillary lymph node dissection (32). Long-term follow-up data from Fitzsullivan et al. have showed no difference in LRR among patients receiving SLNB alone, PMRT alone and both ALND and PMRT (46). Therefore, more prospective studies are needed to evaluate the need for PMRT in patients with sentinel lymph node micrometastatic breast cancer.

Throughout the included studies, there are some potential sources of heterogeneity that need to be explained. First, although most included studies have similar definitions of survival endpoints, the follow-up time varies greatly among different studies. This may result in differences in the observed endpoint events. Second, the number and sample size of studies with different survival endpoints vary greatly, leading to differences in the reliability of results for different survival endpoints. Therefore, it is still necessary to conduct prospective multicenter randomized controlled trials in the future to confirm the application value of PMRT. Third, from a clinical perspective, there is a relationship between axillary lymph node micrometastasis and local recurrence. Therefore, the scope of axillary lymph node dissection may significantly affect survival outcomes. However, due to the limitations of these studies included, we are unable to evaluate the impact of axillary lymph node dissection scope on survival endpoints. Finally, the literature included in this study has a wide range of publication years, which means that there are significant differences in radiotherapy techniques, and different radiotherapy techniques may have an impact on the prognosis of cancer. Looking ahead, more prospective studies are needed to further explore the value of PMRT in BCLNMM. In addition, more innovative radiotherapy techniques may bring smaller treatment side effects, improve treatment accuracy, and have the potential to improve prognosis.

There are some limitations in our study that need to be discussed. First of all, all the studies included in this study are retrospective clinical studies. Therefore, bias associated with retrospective studies is unavoidable. Second, some of the studies were based on SEER databases, which lacked specific available treatment information, which had a certain impact on the survival outcome of patients. Third, the follow-up time of different studies varied greatly, and there may be heterogeneity in the outcome evaluation of survival. Finally, LRRFS and DMFS were only reported in one literature, so meta-analysis could not be conducted.

## 5 Conclusion

In conclusion, despite the tendency of PMRT in BCLNMM to improve DFS, OS, BCSS, LRRFS, and DMFS showed no benefit, therefore, PMRT should be used with caution in BCLNMM. In high-risk populations, more prospective RCTs with long-term follow-up are needed to provide better evidence to guide clinical treatment of PMRT in BCLNMM.

## Data Availability

The original contributions presented in the study are included in the article/**Supplementary Material**. Further inquiries can be directed to the corresponding author.

## References

[1] Łukasiewicz S Czeczelewski M Forma A Baj J Sitarz R Stanisławek A . Breast cancer-epidemiology, risk factors, classification, prognostic markers, and current treatment strategies-an updated review. Cancers (Basel). (2021) 13:1–30. doi: 10.3390/cancers13174287 PMC8428369 34503097

[2] Wilkinson L Gathani T . Understanding breast cancer as a global health concern. Br J Radiol. (2022) 95:20211033. doi: 10.1259/bjr.20211033 34905391 PMC8822551

[3] Sung H Ferlay J Siegel RL Laversanne M Soerjomataram I Jemal A . Global cancer statistics 2020: GLOBOCAN estimates of incidence and mortality worldwide for 36 cancers in 185 countries. CA Cancer J Clin. (2021) 71:209–49. doi: 10.3322/caac.21660 33538338

[4] Cao W Chen HD Yu YW Li N Chen WQ . Changing profiles of cancer burden worldwide and in China: a secondary analysis of the global cancer statistics 2020. Chin Med J (Engl). (2021) 134:783–91. doi: 10.1097/cm9.0000000000001474 PMC8104205 33734139

[5] Xia C Dong X Li H Cao M Sun D He S . Cancer statistics in China and United States, 2022: profiles, trends, and determinants. Chin Med J (Engl). (2022) 135:584–90. doi: 10.1097/cm9.0000000000002108 PMC8920425 35143424

[6] Jacobs AT Martinez Castaneda-Cruz D Rose MM Connelly L . Targeted therapy for breast cancer: An overview of drug classes and outcomes. Biochem Pharmacol. (2022) 204:115209. doi: 10.1016/j.bcp.2022.115209 35973582

[7] Giuliano AE Edge SB Hortobagyi GN . Eighth edition of the AJCC cancer staging manual: breast cancer. Ann Surg Oncol. (2018) 25:1783–5. doi: 10.1245/s10434-018-6486-6 29671136

[8] Shimazu K Noguchi S . Clinical significance of breast cancer micrometastasis in the sentinel lymph node. Surg Today. (2016) 46:155–60. doi: 10.1007/s00595-015-1168-5 25893770

[9] Guani B Mahiou K Crestani A Cibula D Buda A Gaillard T . Clinical impact of low-volume lymph node metastases in early-stage cervical cancer: A comprehensive meta-analysis. Gynecol Oncol. (2022) 164:446–54. doi: 10.1016/j.ygyno.2021.12.015 34949436

[10] Hetterich M Gerken M Ortmann O Inwald EC Klinkhammer-Schalke M Eggemann H . Adjuvant chemotherapy for breast cancer patients with axillary lymph node micrometastases. Breast Cancer Res Treat. (2021) 187:715–27. doi: 10.1007/s10549-021-06162-2 33721148

[11] Mok CW Lai HW . Endoscopic-assisted surgery in the management of breast cancer: 20 years review of trend, techniques and outcomes. Breast. (2019) 46:144–56. doi: 10.1016/j.breast.2019.05.013 31176887

[12] Zhou Y Pu S Jiang S Li D Li S Liu Y . The prognostic significance of further axillary dissection for sentinel lymph node micrometastases in female breast cancer: A competing risk analysis using the SEER database. Front Oncol. (2022) 12:1012646. doi: 10.3389/fonc.2022.1012646 36465338 PMC9713815

[13] Andersson Y Bergkvist L Frisell J de Boniface J . Omitting completion axillary lymph node dissection after detection of sentinel node micrometastases in breast cancer: first results from the prospective SENOMIC trial. Br J Surg. (2021) 108:1105–11. doi: 10.1093/bjs/znab141 34010418

[14] Ren F Yang C Liu J Feng K Shang Q Kang X . An exploratory study of whether axillary lymph node dissection can be avoided in breast cancer patients with positive lymph nodes. Transl Cancer Res. (2024) 13:935–51. doi: 10.21037/tcr-23-1639 PMC10928607 38482409

[15] Man V Wong TT Co M Suen D Kwong A . Sentinel lymph node biopsy in early breast cancer: magnetic tracer as the only localizing agent. World J Surg. (2019) 43:1991–6. doi: 10.1007/s00268-019-04977-1 30888473

[16] Luo S Fu W Lin J Zhang J Song C . Prognosis and local treatment strategies of breast cancer patients with different numbers of micrometastatic lymph nodes. World J Surg Oncol. (2023) 21:202. doi: 10.1186/s12957-023-03082-x 37430331 PMC10332040

[17] Chun JW Kim J Chung IY Ko BS Kim HJ Lee JW . Sentinel node biopsy alone for breast cancer patients with residual nodal disease after neoadjuvant chemotherapy. Sci Rep. (2021) 11:9056. doi: 10.1038/s41598-021-88442-x 33907236 PMC8079673

[18] Dosani M Truong PT . Controversies in locoregional management of breast cancer with low volume pN0(i+) and pN1mi nodal disease. Expert Rev Anticancer Ther. (2019) 19:803–10. doi: 10.1080/14737140.2019.1660165 31498712

[19] Garcia-Tejedor A Ortega-Exposito C Salinas S Luzardo-González A Falo C Martinez-Pérez E . Axillary lymph node dissection versus radiotherapy in breast cancer with positive sentinel nodes after neoadjuvant therapy (ADARNAT trial). Front Oncol. (2023) 13:1184021. doi: 10.3389/fonc.2023.1184021 37621686 PMC10446877

[20] Bartels SAL Donker M Poncet C Sauvé N Straver ME van de Velde CJH . Radiotherapy or surgery of the axilla after a positive sentinel node in breast cancer: 10-year results of the randomized controlled EORTC 10981–22023 AMAROS trial. J Clin Oncol. (2023) 41:2159–65. doi: 10.1200/jco.22.01565 36383926

[21] Moher D Liberati A Tetzlaff J Altman DG Group P . Preferred reporting items for systematic reviews and meta-analyses: the PRISMA statement. J Clin Epidemiol. (2009) 62:1006–12. doi: 10.1016/j.jclinepi.2009.06.005 19631508

[22] Higgins JP Altman DG Gøtzsche PC Jüni P Moher D Oxman AD . The Cochrane Collaboration’s tool for assessing risk of bias in randomised trials. BMJ. (2011) 343:d5928. doi: 10.1136/bmj.d5928 22008217 PMC3196245

[23] Stang A . Critical evaluation of the Newcastle-Ottawa scale for the assessment of the quality of nonrandomized studies in meta-analyses. Eur J Epidemiol. (2010) 25:603–5. doi: 10.1007/s10654-010-9491-z 20652370

[24] Parmar MK Torri V Stewart L . Extracting summary statistics to perform meta-analyses of the published literature for survival endpoints. Stat Med. (1998) 17:2815–34. doi: 10.1002/(SICI)1097-0258(19981230)17:24<2815::AID-SIM110>3.0.CO;2-8 9921604

[25] Chang JS Lee J Kim KH Sohn JH Kim SI Park BW . Do recent advances in diagnostic and therapeutic procedures negate the benefit of postmastectomy radiotherapy in N1 patients with a low risk of locoregional recurrence? Med (Baltimore). (2015) 94:e1259. doi: 10.1097/md.0000000000001259 PMC4616432 26287410

[26] Eastwick G Daugherty M Bogart J Shapiro A . Postmastectomy radiation leads to increased mortality in patient with nodal micrometastasis. Radiother Oncol. (2018) 127:S382–S3. doi: 10.1016/S0167-8140(18)31055-7

[27] Picado O Khazeni K Allen C Yakoub D Avisar E Kesmodel SB . Extent of regional lymph node surgery and impact on outcomes in patients with early-stage breast cancer and limited axillary disease undergoing mastectomy. Breast Cancer Res Treat. (2018) 171:461–9. doi: 10.1007/s10549-018-4840-9 29869773

[28] Wu SP Tam M Shaikh F Lee A Chun J Schnabel F . Post-mastectomy radiation therapy in breast cancer patients with nodal micrometastases. Ann Surg Oncol. (2018) 25:2620–31. doi: 10.1245/s10434-018-6632-1 29987606

[29] Zhang WW Tong Q Sun JY Hua X Long ZQ Deng JP . 21-gene recurrence score assay could not predict benefit of post-mastectomy radiotherapy in T1–2 N1mic ER-positive HER2-negative breast cancer. Front Oncol. (2019) 9:270. doi: 10.3389/fonc.2019.00270 31041190 PMC6477026

[30] Patel M Li C Aronson JH Howie CM Maraboyina S Prabhu AV . The effect of post mastectomy radiation therapy on survival in breast cancer patients with N1mic disease. Breast. (2020) 51:50–6. doi: 10.1016/j.breast.2020.02.009 PMC7375567 32213441

[31] Shi J Lian CL Chi F Zhou P Lei J Hua L . Prognostic and predictive value of the american joint committee on cancer pathological prognostic staging system in nodal micrometastatic breast cancer. Front Oncol. (2020) 10:570175. doi: 10.3389/fonc.2020.570175 33392071 PMC7775531

[32] Lim SZ Kusumawidjaja G Mohd Ishak HM Tan BKT Tan SY Hamzah JL . Outcomes of stage I and II breast cancer with nodal micrometastases treated with mastectomy without axillary therapy. Breast Cancer Res Treat. (2021) 189:837–43. doi: 10.1007/s10549-021-06341-1 34342766

[33] Luo H Yang OO He JL Lan T . Impact of post-mastectomy radiation therapy for sentinel lymph node micrometastases in early-stage breast cancer patients. Med Sci Monit. (2022) 28:e933275. doi: 10.12659/msm.933275 35094003 PMC8812040

[34] Balla A Weaver DL . Pathologic evaluation of lymph nodes in breast cancer: contemporary approaches and clinical implications. Surg Pathol Clin. (2022) 15:15–27. doi: 10.1016/j.path.2021.11.002 35236631

[35] Akezaki Y Nakata E Kikuuchi M Tominaga R Kurokawa H Hamada M . Risk factors for early postoperative psychological problems in breast cancer patients after axillary lymph node dissection. Breast Cancer. (2020) 27:284–90. doi: 10.1007/s12282-019-01020-y 31679113

[36] Jafferbhoy S McWilliams B . Clinical significance and management of sentinel node micrometastasis in invasive breast cancer. Clin Breast Cancer. (2012) 12:308–12. doi: 10.1016/j.clbc.2012.07.012 23039999

[37] Kunkler IH Chua BH . Postmastectomy radiotherapy: a review. Curr Opin Oncol. (2021) 33:547–52. doi: 10.1097/cco.0000000000000789 34494609

[38] Biancosino A Bremer M Karstens JH Biancosino C Meyer A . Postoperative periclavicular radiotherapy in breast cancer patients with 1–3 positive axillary lymph nodes. Outcome and morbidity. Strahlenther Onkol. (2012) 188:417–23. doi: 10.1007/s00066-012-0083-6 22410836

[39] Galimberti V Cole BF Viale G Veronesi P Vicini E Intra M . Axillary dissection versus no axillary dissection in patients with breast cancer and sentinel-node micrometastases (IBCSG 23-01): 10-year follow-up of a randomised, controlled phase 3 trial. Lancet Oncol. (2018) 19:1385–93. doi: 10.1016/s1470-2045(18)30380-2 30196031

[40] Giuliano AE Hunt KK Ballman KV Beitsch PD Whitworth PW Blumencranz PW . Axillary dissection vs no axillary dissection in women with invasive breast cancer and sentinel node metastasis: a randomized clinical trial. JAMA. (2011) 305:569–75. doi: 10.1001/jama.2011.90 PMC5389857 21304082

[41] Solá M Alberro JA Fraile M Santesteban P Ramos M Fabregas R . Complete axillary lymph node dissection versus clinical follow-up in breast cancer patients with sentinel node micrometastasis: final results from the multicenter clinical trial AATRM 048/13/2000. Ann Surg Oncol. (2013) 20:120–7. doi: 10.1245/s10434-012-2569-y 22956062

[42] Darby S McGale P Correa C Taylor C Arriagada R Clarke M . Effect of radiotherapy after breast-conserving surgery on 10-year recurrence and 15-year breast cancer death: meta-analysis of individual patient data for 10,801 women in 17 randomised trials. Lancet. (2011) 378:1707–16. doi: 10.1016/s0140-6736(11)61629-2 PMC3254252 22019144

[43] Gupta N Chugh Y Chauhan AS Pramesh CS Prinja S . Cost-effectiveness of Post-Mastectomy Radiotherapy (PMRT) for breast cancer in India: An economic modelling study. Lancet Reg Health Southeast Asia. (2022) 4:100043. doi: 10.1016/j.lansea.2022.100043 37383992 PMC10306019

[44] (EBCTCG) EBCTCG . Long-term outcomes for neoadjuvant versus adjuvant chemotherapy in early breast cancer: meta-analysis of individual patient data from ten randomised trials. Lancet Oncol. (2018) 19:27–39. doi: 10.1016/s1470-2045(17)30777-5 29242041 PMC5757427

[45] Everett AS De Los Santos JF Boggs DH . The evolving role of postmastectomy radiation therapy. Surg Clin North Am. (2018) 98:801–17. doi: 10.1016/j.suc.2018.03.010 30005775

[46] FitzSullivan E Bassett RL Kuerer HM Mittendorf EA Yi M Hunt KK . Outcomes of sentinel lymph node-positive breast cancer patients treated with mastectomy without axillary therapy. Ann Surg Oncol. (2017) 24:652–9. doi: 10.1245/s10434-016-5605-5 PMC5977973 27822630

